# Adult urethral stricture: practice of Turkish urologists

**DOI:** 10.1590/S1677-5538.IBJU.2014.0672

**Published:** 2016

**Authors:** Mehmet Akyuz, Zulfu Sertkaya, Orhan Koca, Selahattin Calıskan, Musab Ali Kutluhan, Muhammet Ihsan Karaman

**Affiliations:** 1 Department of Urology, Haydarpasa Numune Training and Research Hospital, Istanbul, Turkey

**Keywords:** Urethra, Therapeutics, Pathology

## Abstract

**Objectives:**

To evaluate national practice patterns in the treatment of male anterior urethral strictures among Turkish urologists.

**Materials and Methods:**

A survey form including 12 questions prepared to determine active Turkish urologists' approach to diagnosis and treatment of the adult urethral stricture (US) were filled out. Based on the survey results, the institutions which 218 urologists work and their years of expertise, methods they used for diagnosis and treatment, whether or not they perform open urethroplasty and timing of open urethroplasty were investigated.

**Results:**

Optic internal urethrotomy and dilatation are the most commonly used minimal invasive procedures in treatment of US with the ratios of 93.5% and 63.3% respectively. On the other hand it was seen that urethroplasty was a less commonly used procedure, compared to minimal invasive techniques, with the ratio of 36.7%. Survey results showed us that the number of US cases observed and open urethroplasty procedures performed increases with increasing years of professional experience.

**Conclusions:**

As a method demanding special surgical experience and known as a time-consuming and challenging procedure, open urethroplasty will be able to take a greater part in current urological practice with the help of theoretical education and practical courses given by specific centers and experienced authors.

## INTRODUCTION

Urethral stricture is a well-known and common problem in pathology which, as the result of scar formation, causes stenosis of urethral lumen in the subepithelial tissue (1). Even though treatment of urethral stricture due to different etiological reasons keeps improving, it is still one of the most challenging and complex issues in urological practice (2). Different minimally invasive procedures like periodic dilatation, direct visual internal urethrotomy (DVIU) and urethral stent placement are commonly applied forms of treatment. The minimally invasive procedure is easy to perform and also, this method can be used more than once on the same patient. The fact that results, in the long term, have not shown to be satisfactory has made definitive urethroplasty a necessity (3, 4).

Although it is a method which is technically more complicated and needs expertise, open urethroplasty is a method which has been effective over the last century with remarkably high success rates (5). While long-term success of minimally invasive surgery is about 50%, the success of open urethroplasty is over 90% (6, 7). As there is no consensus of the best form of treatment of urethral stricture, urologists seem to have doubts in deciding whether to use the minimally invasive procedure which is an easy method of treatment that has shown to be less effective in the long run, or open urethroplasty which is a more complex and time consuming procedure, but has excellent long term results.

As a consequence, various studies show that different approaches can be seen on a national and international level in diagnosis, treatment and patient follow-up in cases of urethral stricture (8–10). In this study our aim is to define which techniques are used by Turkish urologists in the process of diagnosis and treatment of urethral stricture, how often open urethroplasty and minimally invasive methods are used and the relationship of choice of treatment with the institutions the urologists work for.

## MATERIALS AND METHODS

On the 22nd National Congress of Urology in Turkey in 2012, a survey called “Approach to urethral stricture survey form” consisting of 12 questions (Table-1), prepared to define approaches of Turkish urologists to diagnosis and treatment of urethral stricture, was given to participants to be filled out. 274 participants who filled the form were included in the study. 56 participants were excluded as some of them were still residents and some experts were also excluded due to the fact that their survey forms were not filled out completely.

**Table 1:** survey form.

Survey form for Approaching Urethral Structure
**1) Mark the institution you work for.**
**a)** University Hospital **b)** Training and Research Hosp. **c)** Other
**2) How many years have you worked in your field of experience?**
**a)** 1-3 y **b)** 4-6 y **c)** 7-9 y **d)** >10 y
**3) How many patients with urethral stricture problems did you treat in 1 year?**
**a)** 1-5 **b)** 6-10 **c)** 11-20 **d)** >20
**4) How do you diagnose urethral stricture? (more than one answer is possible)**
**a)** Retrograd - anterograd urethrography **b)** Urethrocystoscopy **c)** Uroflowmetry **d)** USG **e**) Other
**5) Which technique do you use for the treatment? (more than one answer is possible)**
**a)** Dilatation **b)** Optical internal urethrotomy **c)** Open urethroplasty **d)** Urethral stent
**e**) Perineal urethrostomy
**6) How do you specify the indication of open urethroplasty?**
**a)** Age of the patient **b)** Length and localization of the structure
**c)** Number of the previous urethral structure operations d) Failure of other techniques **e**) All
**7) When do you perform / direct the patient to open urethroplasty?**
**a)** First diagnosis of the structure **b)** First failure of internal urethrotomy
**c)** Second failure of internal urethrotomy **d)** Third failure of internal urethrotomy **e**) Never
**8) Do you perform open urethroplasty?**
**a)** No **b)** Yes
**9) Which technique do you use in open urethroplasty? (more than one answer is possible)**
**a)** End to end anastomosis **b)** Dermal graft **c)** Buccal mucosal graft
**10) When did you perform your last open urethroplasty?**
**a)** Last month **b)** In this year **c)** A few years ago **d)** Don't remember **e**) I refer these patients
**11) Do you think open urethroplasty is ignored in Turkey?**
**a)** Yes **b)** No **c)** No idea
**12) If your answer is yes to Q11, the reason for this can be:**
**a)** Open urethroplasty is a hard procedure.
**b)** The specialists lack experience.
**c)** Endoscopic surgery is more applicable and has better outcomes.
**d)** The open urethroplasty treatment rate is low.

The 218 urologists who were a part of this study represent 15% of all urologists in Turkey. The results of this survey have revealed relevant information about the 218 urologists who have taken part in this study. The results show which institutions the urologists work for and also give information about their years of expertise. Furthermore, data could be found on the methods used by the participants for diagnosing and forms of treatment used. Also, the results present us with information on whether the participants performed open urethroplasty or not, in which circumstances they tend to use open urethroplasty and the reasons for ruling it out. Nevertheless, urethroplasty rates were checked upon and research was done to get information about the impact that institutions have on the decisions which the urologists have to make when it comes to the method of treatment when they choose to perform surgery.

For statistical analysis, the computer programme SPSS® for Windows was used. The Student T test and Mann Whitney U test were used for data analysis. A P value<0.05 was considered statistically significant.

## RESULTS

The results of the study are summarized in Table-2. According to the survey results, the most commonly used methods in the diagnosis of urethral stricture in Turkey were urethrocystoscopy (85.3%), uroflowmetry (69.7%) and retrograde urethrography (55.0%). Ultrasound and urethral calibration were rarely used methods for the diagnosis of urethral stricture (0.9%).

**Table 2:** survey Results.

Variables		No (%)
**Institute**		
	Training-research hospital	58 (26.6)
	University hospital	60 (27.5)
	Other (Private - State hospital)	100 (47.9)
**Professional Experience**		
	1-3 y	34 (15.6)
	4-6 y	20 (9.2)
	7-9 y	50 (22.9)
	10 y and above	114 (52.3)
**Urethral structure treatment per year**		
	1-5 cases	12 (5.5)
	6-10 cases	26 (12.9)
	11-19 cases	70 (32.1)
	20 and above cases	110 (50.5)
**Methods for Diagnosis**		
	Retrograde urethrography	120 (55.0)
	Urethrocystoscopy	186 (85.3)
	Uroflowmetry	152 (69.7)
	USG and other	2 (0.9)
**Methods for Treatment**		
	Urethral dilatation	138 (63.3)
	Internal urethrotomy	204 (93.5)
	Urethroplasty	80 (36.7)
**End-to end anostomosis**		80 (36.7)
	Buccal graft	48 (22)
	Dermal graft	22 (10)
	Urethral stent	18 (8.2)
	Perineostomy	8 (3.7)
**Perform open urethroplasty?**		
	Yes	80 (36.7)
	No	138 (63.3)
**Time of referral to open urethroplasty**		
First diagnosis		2 (0.9)
	After 1^st^ failure	12 (5.6)
	After 2^nd^ failure	72 (33.0)
	After 3^rd^ failure	122 (55.9)
Never		10 (4.6)
**Is open urethroplasty ignored?**		
	Yes	168 (77.0)
	No	26 (11.9)
	No idea	24 (11.1)
**Reasons for ignoring open urethroplasty**		
	Difficulty	94 (43.1)
	Lack of experience	144 (66.0)
	Endoscopic procedures are easier and more successful	100 (45.8)
	Urethroplasty lacks success	30 (13.7)

Optic internal urethrotomy and dilatation are the most commonly used minimally invasive procedures in treatment of urethral stricture with the ratios of 93.5% and 63.3% respectively. On the other hand, urethroplasty with the ratio of 36.7% is a less common procedure, compared to other minimally invasive techniques. According to the results of the survey, 63.3% of the urologists stated that they did not perform open urethroplasty while 8.2% of the participants used urethral stents and 3.7% performed perineal urethrostomy. The survey results showed that as the physician's years of experience increase, so does the number of open urethroplasty procedures that they perform in cases of urethral stricture.

When the institution that employs the urologist who performs open urethroplasty procedures is taken into consideration, it can be said that no statistically significant differences were seen between training-research hospitals and university hospitals. On the other hand, statistically significant differences were seen when training-research hospitals and university hospitals were compared with other hospitals. Results of the evaluation of minimally invasive methods used in hospitals show that training-research hospitals and university hospitals had similar results in contrast to other hospitals where the methods were applied at a significantly higher rate. When techniques of open urethroplasty are compared, it can be said that the difference between research-training hospitals and university hospitals were statistically significant (Table-3).

**Table 3:** procedures in urethral stricture treatment according to the institutions.

		Training and Research Hosp No (%)	University Hospital No (%)	Other Hospital No (%)	P Value
Doing urethroplasty or not	Yes	30 (37.5)	34 (42.5)	16 (20.0)	P_1_:0.20
					P_2_:0.002
	No	28 (20.3)	22 (15.9)	88 (63.8)	P_3_:0.001
					P_1_:0.32
	Optic internal urethrotomy	56 (27.4)	54 (26.4)	94 (46.0)	P_2_:0.001
					P_3_:0.001
					P_1_:0.08
Procedure	Dilatation	30 (21.7)	40 (28.9)	68 (49.2)	P_2_:0.001
					P_3_:0.001
					P_1_:0.20
	Urethroplasty	30 (37.5)	34 (42.5)	16 (20.0)	P_2_:0.002
					P_3_:0.001
					P_1_:0.05
	End to end anastomosis	28 (34.7)	33 (41.3)	19 (23.9)	P_2_:0.018
					P_3_:0.001
					P_1_:0.046
Methods for urethroplasty	Buccal mucosal graft	18 (37.5)	22 (45.8)	8 (16.7)	P_2_:0.01
					P_3_:0.001
					P_1_:0.10
	Dermal graft	12 (54.5)	10 (45.5)	0 (0.0)	P_2_<0.001
					P_3_<0.001

**p**_1_ = Training-research hospitals – University hospitals

**p**_2_ = Training-research hospitals – Other hospitals

**p**_3_ = University hospitals – Other hospitals

## DISCUSSION

Urethral stricture has been known for a long time, the symptoms differ from a mild decrease in urine flow velocity to inability to void and as it has a high recurrence rate after treatment it is considered a serious urological problem (1–3). Within the last 30 years, a significant change in the treatment algorithm of urethral stricture, caused by trauma, infection, idiopathic reasons and iatrogenic reasons like transurethral surgery, catheterization and cystoscopy, was observed from more frequently performed minimally invasive techniques to definitive urethroplasty (4, 5).

In van Leeuwen et al.'s research from the Netherlands, 72% of the urologists use urethrography in the diagnosis of urethral structure, on the other hand, urethrography use in Palminteri et al. research is at a surprisingly low rate of 16% indicating the denial of the test by the Italian patients and acceptance of the technique as invasive (9, 10). It is observed that ultrasonography is seldom used in the diagnosis of urethral stricture (0.9%) but uroflowmetry (69.2%) and urethrocystoscopy (85.3%) is frequently used. In our study we have observed that the ratio of using retrograde urethrography, which is the basic assessment method, is 55%.

Firstly described by Sachse in 1974, cold––cut direct visual internal urethrotomy (DVIU) is a popular method that is used frequently, easily and safely in current urological practices (11). According to Pansadoro and Heyns's studies, recurrence ratios after the first, second and third DVIU, are 61%, 100% and 100% respectively in a 4 year patient follow-up (4, 12). In a prospective, randomized study that compares optical urethrotomy to dilation, no significant difference was shown between both methods in the aspect of the recurrence of the stenosis (13). According to our results, the most frequent methods used for adult urethral stricture by urologists in Turkey are minimally invasive methods such as DVIU and urethral dilatation. Urologist that took the survey indicated that they use DVIU (93.5%) and dilatation (63.3%). These results are similar with the national survey results of the USA, the Netherlands and Italy (8–10).

Our observations showed that 8.2% of urologists use urethral stents which is associated with restenosis and complications, and which fails to show long-term effects. Urethral stent usage is 1.3% in van Leeuwen's study, 12% in Palminteri's and 23.4% in Bullock's study (14, 15). The perineostomy implementation ratio is 3.7% and it shows similarity with the other survey results.

Open urethroplasty is the best and most definitive surgical technique in strictures in which minimally invasive techniques failed: cases with complete obstruction, long and multiple strictures and in serious spongiofibrosis. This technique which requires technical skills and good experience has a success rate of about 90-95% and a recurrence rate of 2-7% (16, 17). 36.7% of our survey participants perform open urethroplasty. In other studies this rate is about 29-42% (8–10). According to four different national survey studies, there are no serious differences in diagnosis and treatment of urethral stricture among urologists. When we compared the techniques used in open urethroplasty, we saw that end-to-end anastomosis was more frequently used than graft techniques. The reason for more frequent use of end-to-end anastomosis rather than graft techniques may be not combining a complicated procedure which requires special surgical experience, special care and special instrumentation with another complicated procedure.

One of the interesting results of our study is the relationship between the institution the treating physician works for and the treatment techniques for urethral stricture. When we compared the use of open urethroplasty and minimally invasive techniques between university hospitals and training and research hospitals where academic urologists work, there was no significant difference. On the other hand, there was a significant difference between them when compared with urologists working in other hospitals. Another result of our study is that 77% of urologists ignored open urethroplasty and the main reason for this was a lack of experience (66%). For this reason, just as stated in the study of Bullock et al., due to financial worries and concerns minimally invasive procedures are performed more frequently than definitive urethroplasty (8).

Another interesting result of our study was that open urethroplasty is generally preferred after two to three unsuccessful DVIUs in urethral stricture. A rate of 0.9% in preferring open urethroplasty in the first diagnosis makes us think that primarily minimally invasive techniques are preferred in almost all urethral strictures. However, according to literature in this field, multiple dilatations and internal urethrotomy procedures lower the success rate of urethroplasty and affect the results negatively. Updated survey results show that urologists lead their patients to urethroplasty after the 2^nd^ and 3^rd^ failure (75% in the study of Bullock et al. and 88.9% in our study (8, 18, 19).

A relatively low number of participants and the preference of the urologists who attended the congress can be seen as the limitations of our research. Likewise, the usage of an invalid form has been another limitation for our research.

## CONCLUSIONS

Minimally invasive methods are the most frequently used procedures in the treatment of urethral stricture in Turkey as observed in the survey results. These methods became popular because they are easily applicable and show good results in short term, while on the other hand, in the long term, stricture relapse is the most important flaw. End-to-end anastomosis and urethroplasty with buccal graft is less frequently used. Open urethroplasty, which is a method demanding special surgical experience and is known as a time-consuming and challenging procedure, can be more frequently performed in the current urological practice with the help of theoretical education and practical courses given by specific centres and experienced authors.
